# Entropy Generation Methodology for Defect Analysis of Electronic and Mechanical Components—A Review

**DOI:** 10.3390/e22020254

**Published:** 2020-02-23

**Authors:** Miao Cai, Peng Cui, Yikang Qin, Daoshuang Geng, Qiqin Wei, Xiyou Wang, Daoguo Yang, Guoqi Zhang

**Affiliations:** 1 School of Mechanical and Electrical Engineering, Guilin University of Electronic Technology, Guilin 541004, China; pengcui618@163.com (P.C.); qykbeicheng97@163.com (Y.Q.); gengdaoshuang@163.com (D.G.); weiqiqin1992@163.com (Q.W.); wxy_07@126.com (X.W.); g.q.zhang@tudelft.nl (G.Z.); 2 Delft Institute of Microsystems and Nanoelectronics (Dimes), Delft University of Technology, 2628CD Delft Mekelweg 6, The Netherlands

**Keywords:** entropy generation, methodology, electronic and mechanical components, defect, diagnosis, reliability

## Abstract

Understanding the defect characterization of electronic and mechanical components is a crucial step in diagnosing component lifetime. Technologies for determining reliability, such as thermal modeling, cohesion modeling, statistical distribution, and entropy generation analysis, have been developed widely. Defect analysis based on the irreversibility entropy generation methodology is favorable for electronic and mechanical components because the second law of thermodynamics plays a unique role in the analysis of various damage assessment problems encountered in the engineering field. In recent years, numerical and theoretical studies involving entropy generation methodologies have been carried out to predict and diagnose the lifetime of electronic and mechanical components. This work aimed to review previous defect analysis studies that used entropy generation methodologies for electronic and mechanical components. The methodologies are classified into two categories, namely, damage analysis for electronic devices and defect diagnosis for mechanical components. Entropy generation formulations are also divided into two detailed derivations and are summarized and discussed by combining their applications. This work is expected to clarify the relationship among entropy generation methodologies, and benefit the research and development of reliable engineering components.

## 1. Introduction

Entropy generation is a physical quantity representing energy loss, and it can quantify energy transmission and judge thermal performance. The heat flux density of chips has increased with the miniaturization of power devices. The continuous high heat injection causes heat concentration and defects at critical interfaces. High power density and high operating temperature require electronic and mechanical components to have high mechanical and thermal performance [1]. Several technologies for determining reliability, such as thermal modeling, cohesion modeling, statistical distribution, and entropy generation analysis, have been developed. Thermal modeling is useful in analyzing the heat transfer performance of light-emitting diodes (LEDs) [2,3] and insulated-gate bipolar transistors [4] as it reveals junction temperature, thermal conductivity, and thermal resistance [5,6]. However, temperature and thermal resistance are limited in characterizing energy transmission states systematically.

The aging process is generally regarded as a cumulative result of irreversible thermodynamics [7]. Dissipative processes, along with a reliable life span, generate irreversible entropy. Entropy generation as an analytical method has attracted increasing attention in many applications, such as electronic devices, mechanical structures, and heat exchangers. Entropy can integrate the thermal-related parameters of an entire system [8]. The thermodynamic law is an important tool that is usually applied to the optimization of cooling system performance. Extensive numerical and theoretical research on entropy generation methodologies has been carried out over the years with the goal of predicting and diagnosing the reliability of electronic or mechanical components. The second law of thermodynamics serves as an indicator of heat transfer performance.

**Figure 1:**
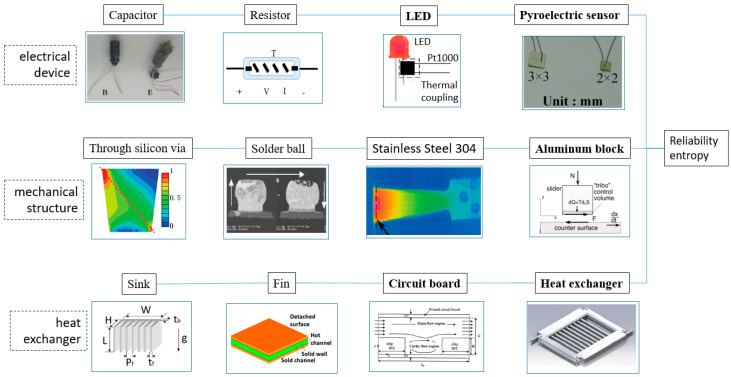
Application of entropy generation in the reliability field. (Capacitor [12], resistor [11], light emitting diode (LED) [13], pyroelectric sensor [30], through silicon via [15], solder ball [9], 304 stainless steel [18], aluminum block [7], sink [25], fin [31], circuit board [22], and heat exchanger).

Entropy generation methodologies have been widely applied in the reliability field. The applications of entropy generation are summarized in Figure 1. First, the failure of electronic devices due to electromigration coupled with thermal migration is analyzed on the basis of entropy theory [9]. The defect prediction of key interfaces, such as die-attach (DA) layers [10], resistors [11], capacitors [12], LEDs [13], and chips, is also investigated. Second, entropy generation on mechanical structures is considered in several defect cases. The destructive stress in critical microstructures can also be predicted with the analysis of entropy generation [14,15]. In addition, structural fatigue and failure in microscopic or macroscopic objectives can be effectively characterized by entropy analysis. For example, the failure life, cyclic energy diffusion, and irreversible energy loss of Al metals have been analyzed [16,17,18]. Existing theories show that entropy generation failure can help analyze the failure attributes of components, such as the failure cycle period and unit cycle energy loss [17,19]. The analysis of entropy generation plays an important role in characterizing the reliability of electronic or mechanical components. Moreover, improvements in heat exchangers [20], nanofluids, and sinks and their fins [21] require not only heat transfer entropy but also fluid entropy and friction entropy. Previous studies analyzed the structural problems of circuit boards [22], 2D orthotropic convection pin fins [21], plate-fin heat exchangers [23], dual pressure waste heat boilers [24], and sinks [25]. An optimal thermal resistance can help to minimize the junction temperature of a system. The optimization of entropy generation calculation is integrated with the optimization of thermal resistance and indicates an adequate temperature distribution [26,27,28,29].

Characterizing the defects of electronic and mechanical components to diagnose component lifetime systematically has gradually become a hot topic in recent years. The current work aimed to review previous defect analysis studies that used entropy generation methodologies for electronic and mechanical components and to provide a clear theoretical framework and its applications. In this work, entropy generation methodologies are classified into two categories: damage analysis for electronic devices and defect diagnosis for mechanical components. Developed entropy generation formulations are also classified into two detailed derivations and then summarized and discussed by combining their applications. Section 2 and Section 3 of this paper cover the physical aspects of entropy generation in reliable electronic devices and mechanical components, respectively. Section 4 provides conclusions and suggestions for future work.

## 2. Entropy Methodologies for Damage Characterization of Electronic Components

The second law of thermodynamics provides a measure of entropy generation rate and irreversibility within a system or process and thus characterizes the efficiency of the heat transfer process. Clausius proposed the first mathematical expression of entropy as

**Figure 2:**
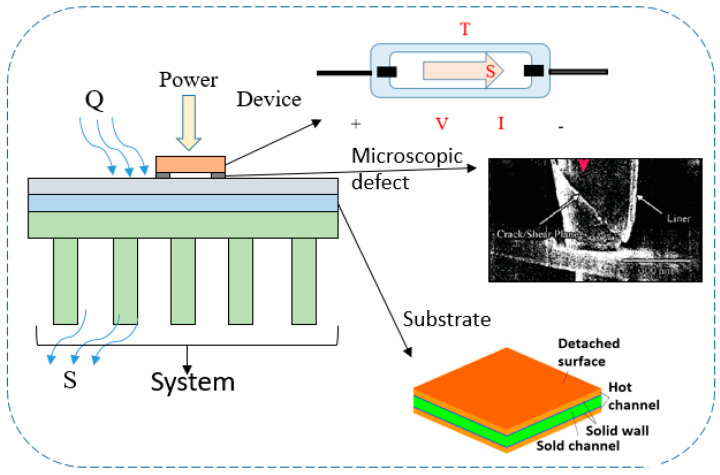
Entropy generation analysis for damage characterization of electronic systems (microscopic defect from [14]).

(1) S=∫ dQ/T

where *S* is the entropy generation, *Q* is the heat, and *T* is the temperature. The following is an introduction to the application and analysis of irreversible properties for evaluating the aging failure of electronic devices. On the basis of the research progress of entropy generation analysis for the damage characterization of electronic systems (Figure 2), existing methodologies are summarized in four parts, namely theories on electronic devices, microstructures, composite boards, and electronic systems.

### 2.1. Entropy Methodologies for Electronic Devices

The entropy generation of commercial 0.25 W carbon film resistors has been analyzed by detecting current, voltage, and temperature [11]. Entropy, a valuable indicator of resistor degradation, is even more important than resistance. Resistance fluctuates because of external factors, including electromigration, mechanical stress-strain, acidification, and oxidation; hence, a theoretical model to understand the relationship between entropy generation and resistor degradation has been proposed [11].

The first law of thermodynamics is expressed as

(2) $$dE_{in}=dW+dQ+dE_{irr}$$

(3) $$E_{in}=\int \;Pdt=\int \;UIdt$$

(4) $$W_{\mathrm{light}}=\int_{0}^{\infty }I_{light}\left(\lambda \right)d\lambda$$

(5) $$Q=R_{s}I^{2}+\frac{U_{D}^{2}}{R_{P}}$$

Entropy balance is provided in the equation of the second law of thermodynamics for intrinsic and external states in a 3D object. Generally, a system with an irreversible process can be expressed as

(6) $$\frac{dS}{dt}=\frac{dS_{e}}{dt}+\frac{dS_{i}}{dt}$$

where the subscripts *e* and *i* refer to external entropy flux and internal entropy generation, respectively.

The entropy generation rate can be described as the input power (*P*) at a certain temperature (*T*). Normally, the internal heat generated during the operation of electronic devices, such as resistors [11], capacitors [12], LEDs [13], is conducted into the external environment. The entropy generation of a resistor is introduced as a typical example below. A general functional relationship among entropy generation value, heat power, current, voltage, and the temperature is expressed as [11]:

(7) $$\frac{dS}{dt}=\frac{P}{T}+\frac{UI}{T}$$

In a continuous bias voltage, an electronic device undergoes heat dissipation until the final heat is balanced to environmental conditions. Formulas (6) and (7) can be combined as follows to express the relationship between irreversible damage entropy and the damage deterioration of power devices [11]:

(8) $$P=UI=Q_{e}+\frac{dS_{i}}{dt}T$$

Therefore, the slope of the linear relationship in Formulas (8) is the irreversible loss entropy generation, and the intercept is the heat dissipated into the environment.

Generally, when an entire system fails due to internal failure or degradation, the relationship between the system failure’s entropy generation rate and the internal failure’s entropy generation rate can be expressed in inequality as

(9) $$\frac{dS}{dt}\gg k_{\lim}\frac{dS_{i}}{dt}$$

where $k_{\lim}$ is a limit constant value regarded as a parameter for diagnosing and predicting damage characteristics. The total entropy generation of the device should be the integral value of the entropy generation rate over time, that is,

(10) $$S=\oint \frac{dSdt}{dt}$$

Entropy generation analysis can determine how much heat power is wasted for the destructive process in a resistor device and is, therefore, important for the assessment of the reliability of electronic devices. Following a similar idea to that for resistors, Cuadras et al. [12] investigated the entropy characterization of overstressed capacitors for lifetime prediction and found that entropy generation was related to capacitor parameters, including capacitance, structural size, and operating voltage. Subsequently, Cuadras et al. [13] carried out a similar investigation into LED lights and determined the entropy generation rate with LED degradation. A thermodynamic damage parameter *S*(*t*) was proposed to project an LED lifetime. The proposed method is promising for avoiding light measurement and failure rate statistics through long-term reliability testing or aging.

**Figure 3:**
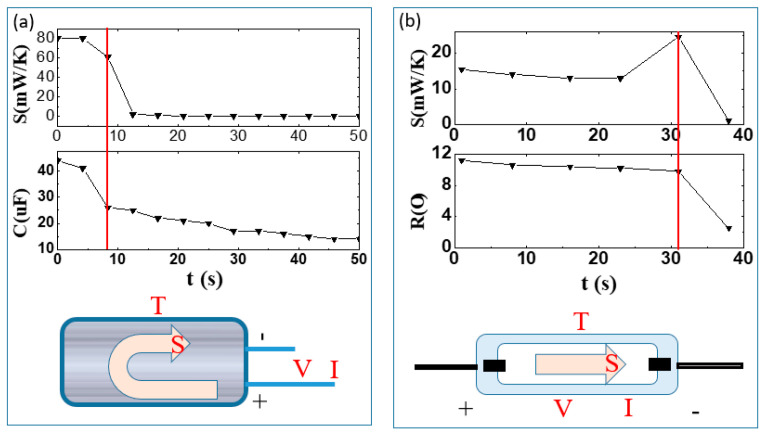
Schematic of monitoring entropy generation rate of capacitor (**a**) [12] and resistor (**b**) [11].

Figure 3 provides a schematic of the entropy generation monitoring of resistors and capacitors [11,12]. In Figure 3a, the capacitor fails with a dramatic drop of its capacitance at approximately 8 s; correspondingly, its entropy generation rate shows an abrupt change at the same moment. Similarly, in Figure 3b, the resistor fails while its resistance declines at approximately 31 s, and the corresponding entropy generation rate sharply decreases to zero at the same time. Evidently, the failure and/or degradation of electronic devices can be characterized effectively by measuring the entropy generation rate.

Hsiao et al. [30] monitored the entropy characteristics of a capacitor with a pyroelectric sensor. The pyroelectric sensor was attached to the capacitor, and the entropy generation rate was detected by sensing the temperature fluctuation, thermal current, and heat capacity of the capacitor in a hot bath. The effect of sensor size on the detection of entropy generation rate was also investigated. The threshold to failure presented on the entropy generation rate curve at different operating voltages could be detected effectively. For the materials of the pyroelectric sensor, the entropy generation rate over time and total entropy generation could be expressed, respectively, as

(11) $$\frac{dS}{dt}=C_{P}I_{P}/(\eta PAT)$$

(12) $$S=\int \;\left(\frac{dS}{dt}\right)dt=\int \;C_{P}\frac{dT}{T}=C_{P}\ln\left(T_{f}/T_{i}\right)$$

The entropy characteristics of a Li-ion battery pack have also been considered in thermal design [32]. An effective thermal model of a Li-ion battery pack has been developed by considering thermodynamic- and transport-related heat sources.

### 2.2. Entropy Methodologies for Microstructures

Changes in microstructures, such as solder joints (eutectic solder ball), microinterconnection structures (through silicon via (TSV)), bonding interfaces, and thin attached layers (DA layers), represent a major concern in electronic devices. Hence, the entropy generation theory, characterizing their behaviors and effects, has been attracting increasing research attention.

Lai et al. [9] performed experimental and theoretical investigations on the microstructure evolution of eutectic SnPb flip-chip solder joints. The effects of entropy generation on the changes in microstructure were investigated by thermomigration. Pb enrichment in the substrate side is mainly caused by a high entropy generation rate in thermomigration. The major entropy generation in irreversible thermomigration is due to heat propagation under a temperature gradient [9]; it is defined as

(13) $$\frac{T}{V}\frac{dS}{dt}=\left(-k\frac{dT}{dx}\right)\left(-\frac{1}{T}\frac{dT}{dx}\right)$$

which agrees with Onsager’s principle of irreversible processes [33]. Sharifah et al. analyzed irreversible thermal migration and electromigration by applying the entropy generation to characterize damage mechanics through a damage parameter (Equations (14)):

(14) $$D=D_{\mathrm{cr}}\left[1-e^{\frac{s_{0}-s}{N_{0}k}}\right]$$

where $N_{0}$ is the Avogadro’s constant, *k* is the Boltzmann’s constant, and *s* is the entropy per unit mass. Then, the thermal migration of a Pb37Sn63 solder joint and the thermal decay process of low carbon steel were investigated.

Wang et al. [15] first applied entropy generation theory to analyze full-chip TSV reliability in an electronic device. The numerical model in this work combined the continuum mechanics law with the thermodynamics law. This model avoided fitted parameters and considered the exhaustive temperature profile *T*(*t*). Entropy generation analysis indicated that the TSV structure was easily influenced on the pad corner than on the pad center under thermal cycles and that a sharp increase in entropy generation at both ends of a diagonal implied a possible crack [15]. The running lifetime of seven central processing unit applications of an Android mobile phone was analyzed in the same way [14].

The traditional cohesive zone model has been used to analyze the effects of delamination propagation behavior on interface heat transfer and plays a key role in interface crack prediction [34]. Extensive research has also attempted to elucidate the relationship between the defects and reliability characteristics of electronic devices, including interface failure effects on reliability [35,36,37], void effects of DA layers in LED packages [38,39,40,41,42,43] and DC-to-DC converters [44]. The degradation of heat transfer performance is one of the main causes of electronic package failure; hence, several studies tried to characterize the mechanism behind the degradation of heat transfer performance from the perspective of energy loss [10,45]. In 1D steady heat conduction, the equation for entropy generation through solids can be expressed as [46]

(15) $$S=\int_{0}^{x}\frac{k}{T^{2}\left(x\right)}{\left(\frac{dT\left(x\right)}{dx}\right)}^{2}dx$$

which takes into account the integral of the temperature distribution and the thermal conductivity on the displacement. This method has been initially applied to evaluate the thermal conductivity of critical interfaces for high-power LEDs [45]. The heat transfer degradation of key interfaces has been studied by presetting the delamination in the edge and center of the DA layer. The dimensionless entropy generation value at the edge position corresponding to the delamination in the DA layer is significantly higher than that at the center position [45]. The evolution mechanism of crack length and the degradation of heat transfer performance have been further considered on the basis of entropy generation theory [15]. The result showed that a large delamination results in the poor heat transfer capability and reliability of devices.

Entropy generation methods in the research of the microstructures of electronic devices are effective but rare, and other methodologies are needed in the future.

### 2.3. Entropy Methodologies for Composite Boards

The thermal reliability of a circuit board carrying many electronic components is extremely important in electronic assembly because of its electrical connection and mechanical protection. Boards with different circuit designs have different thermal conductivities. Bejan et al. [47] studied the minimization of entropy generation in free convection and revealed that the method of entropy generation minimization played an important role in the optimization of the reliability of electronic products. Yang et al. [22] used a minimum entropy generation (MEG) method to optimize the package reliability of multichip boards. They considered two constraints: the highest chip temperature and the limit pressure difference in the channel.

Recently, local entropy generation in composite board systems has been investigated. Local entropy generation is affected by two parameters, namely, position and ratio of internal and external temperatures [46]; the former has the greatest impact. However, the contact interface has been assumed to be the ideal contact interface [46]. The effects of the thermal resistance of the contact interface on entropy generation in asymmetrically cooled composite plates have been studied to meet actual contact interface conditions [26]. The results showed that contact thermal resistance has the greatest influence on the entropy generation of composite geometry.

The comparison between classical entropy generation and MEG for different geometries has been performed [28]. The results showed that the classical and MEG temperature curves are substantially different when both sides of a composite geometry have a strong thermal asymmetry. Meanwhile, the constant conductivities of materials have been compared with temperature-dependent thermal conductivity K(T) and coordinate-dependent thermal conductivity K(x) to study the effects of thermal conductivity on entropy generation [28]. The results showed that temperature-dependent and location-dependent thermal conductivities exert a great influence on entropy generation. Following the study of materials with temperature-dependent properties, slabs with temperature-dependent internal heat generation have been investigated [27,48,49]. The total entropy generation in an internal heating composite board can be minimized by optimizing the temperature ratio on both sides of the composite board.

The work on entropy generation in thermal systems for solid structures has been reviewed in the literature [50]. Accurate numerical methods, rough numerical methods, and software simulations have been also summarized.

### 2.4. Entropy Methodologies for Electronic Systems

Power dissipation in an integrated circuit board is a common and important issue for electronic package designers. The exergy loss of the individual modules of a chip package and the total package has been investigated on the basis of the thermodynamic metric of exergy [51]. Subsequently, a coefficient that measures the uniformity of power distribution and the quantity of power consumption has been added to describe power distribution imaging [30].

The effect of energy transfer has received much attention recently. The exergy theoretical analysis method, which can be applied to predict energy loss in fuel cells and heat engines, has been proposed [52]. The calculation of exergy analysis and entropy generation is essentially an expression of energy loss [50], as provided in Equation (16). The research on the second law of thermodynamics under electronic packaging is limited and is still in its early period. The above literature can be used as theoretical bases for further in-depth systematic research in the future. The modeling process of a flip-chip package system is exemplified in the following to clearly express system-level entropy analysis.

The flow (stream) exergy of a flip-chip package at any point is expressed in Equation (16) [51]. The basic assumption of this method is that the heat transfer on the *Z*-axis is uniform.

(16) $$\psi =\left(G-G_{0}\right)-T_{0}\left(s-s_{0}\right)$$

**Figure 4:**
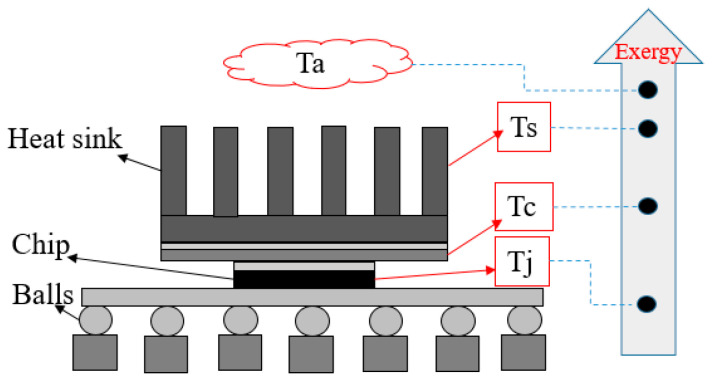
Schematic of exergy diffusion of flip-chip package model.

The thermal resistance of a junction-to-printed circuit board has been ignored. Only three thermal resistances, namely junction-to-case, case-to-sink, and sink-to-ambient resistances, have been considered (Figure 4). Contact and component resistances are included in these quantities. The temperature gradients across each of these resistances can generate exergy; therefore, the total exergy loss in the package can be computed as follows:

(17) $$\psi_{jc}=(\frac{T_{a}}{T_{j}-R_{jc}\cdot \overset{•}{w}})$$

where the junction-to-case exergy loss is presented as

(18) $$\psi_{\mathrm{jc}}=(\frac{T_{a}}{T_{j}-R_{jc}\cdot \overset{•}{w}})$$

where $R_{jc}$ is the junction-to-case resistance.

Case-to-sink exergy is presented as

(19) $$\psi_{cs}=\left(1-\frac{T_{a}}{T_{c}}\right)\overset{•}{w}-\left(1-\frac{T_{a}}{T_{s}}\right)\overset{•}{w}$$

Without the heat sink temperature, the case-to-sink exergy is expressed as

(20) $$\psi_{cs}=\left(\frac{T_{a}}{T_{j}-\left(R_{jc}+R_{cs}\right)\cdot \overset{•}{w}}-\frac{T_{a}}{T_{j}-R_{jc}\cdot \overset{•}{w}}\right)\overset{•}{w}$$

Sink-to-ambient exergy is presented as

(21) $$\psi_{sa}=\left(\frac{R_{sa}\overset{•}{w}}{T_{a}}+\frac{\overset{•}{m_{a}Pr}}{\rho_{a}\cdot \overset{•}{w}}\right)\overset{•}{w}$$

where *R_sa_* is the sink-to-ambient resistance, *T_a_* is the ambient temperature, *P* is the pressure. Herein, the intermediate derivation process based on entropy generation analysis is expressed as

(22) $$S_{gen}=\frac{Q^{2}\cdot R_{sa}}{T_{a}^{2}}+\frac{F_{D}\cdot v_{f}}{T_{a}}$$

Reducing the heat resistance of a radiator to air generally means increasing the efficiency of the second law of thermodynamics. The exergy loss of a chip package can be presented as

(23) $$\psi_{d}=\left(\frac{T_{a}}{T_{c}}\right)\overset{•}{w}+\left(\frac{T_{a}}{T_{s}}-\frac{T_{a}}{T_{c}}\right)\overset{•}{w}+\left(\frac{R_{sa}\overset{•}{w}}{T_{a}}+\frac{\overset{•}{m_{a}\Delta P_{a}}}{\rho_{a}\cdot \overset{•}{w}}\right)\overset{•}{w}$$

A second law efficiency has been proposed to quantify energy efficiency [51]:

(24) $$\eta_{∐}=1-\frac{\Psi_{d}}{\Psi_{in}}=1-\frac{\sum_{n}\sum_{i}{\tilde{R}}_{ni}\cdot {\overset{•}{w}}_{i}}{\sum_{i}{\overset{•}{w}}_{i}}$$

where $\Psi_{d}$ is the energy loss, and $\Psi_{in}$ is the input energy. A high $\eta_{∐}$ indicates low energy loss and high reliability.

The power distribution uniformity on the same board has been quantified using the proposed input uniformity and unevenness coefficient $Z_{power}$ [29]:

(25) $$Z_{power}=\frac{W_{total}-W_{i,max}}{W_{total}-W_{avg}}$$

A large value of $Z_{power}$ indicates that the input power is uniform. Conversely, a small value of $Z_{power}$ indicates that the input power is uneven and impacts the reliability of the entire board. Bejan et al. [47] reviewed the application and development of thermodynamic methods in thermal design from a macro perspective.

Table 1 shows a summary of the above investigations on electronic components based on entropy methodologies.

## 3. Entropy Methodologies for Defect Diagnosis of Mechanical Components

Fatigue is an important indicator of the reliability of mechanical components, including microscopic TSV structures [14] and macroscopic parts of aviation Al [17]. The theory of entropy generation is involved in the diagnosis of the defects of mechanical components and in the subsequent assessment of the reliability of mechanical structures.

### 3.1. Research Progress on Defect Diagnosis

Following the popular stress life (S–N curve) proposed by Wöhler et al. [56], existing studies considered local strain and crack propagation [57]. Meneghetti et al. [58] found that heat dissipation per unit volume per cycle (Qcyc) could be used as a damage parameter in the study of the failure criterion of machinery from the perspective of heat transfer. The fatigue reliability life of samples has been estimated on the basis of experiments [59]. The results showed that Qcyc is an attribute that is independent of operating frequency and sample size.

**Figure 5:**
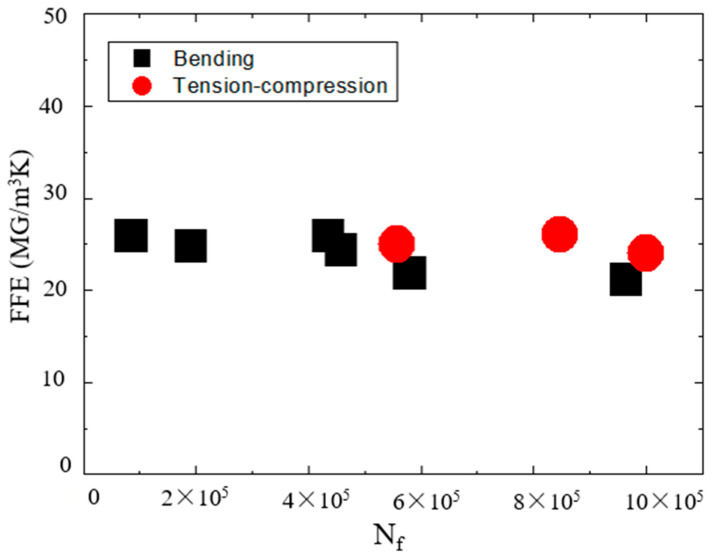
FFE (fracture fatigue entropy) of AISI 1018 carbon steel [64].

However, these articles did not evaluate fatigue reliability from the perspective of energy loss until the work of Italyantsev et al. [60]. The entropy generation rate can be employed in predicting the reliability of a mechanical structure and the service life of its mechanical parts. In service life, the fatigue process produces hysteresis energy [61]. A method for accessing fatigue life by calculating hysteresis energy has been proposed [61]. This approach can simply be promoted to a multiaxial state of work conditions. Following this method, a number of experimental approaches using the second law of thermodynamics for mechanical reliability have been presented [16,17,18,19,62,63]. For example, the fatigue degree and service life of Al and stainless steel have been analyzed with entropy theory [16]. Naderi et al. [17] judged the fatigue of metallic materials on the basis of the irreversibility of cyclic plastic strain energy in the aging process under different loads. Damage thermodynamic entropy can be applied to the evaluation of the degradation behavior of materials. With the continuous improvement of entropy generation theory in mechanical material fatigue, the total entropy from initial fatigue to failure can be regarded as a material property because the total entropy generation is a constant. An example of an aviation material is AA7075-T651 [19]. In other words, the entropy accumulation for break failure life is a constant value in various loads. Similarly, fracture fatigue entropy (FFE) can be calculated on the basis of energy dissipation in the aging process (Figure 5) [64].

A method for evaluating fresh crack surfaces has been proposed on the basis of the macroscopic entropy formula and energy equation to study the damage in isothermal composite materials, such as laminates and sandstone [65]. Apart from the research on composite structures [26,27,28,65], the reliability-entropy hypothesis has been proposed to predict the reliability of mechanical systems [7]. In this approach, system reliability is calculated through the parallel relationships between each subsystem, and the lifetime of the whole mechanical system is assessed on the basis of entropy theory. Eger et al. [62] applied entropy flow and entropy generation theories effectively in an electromechanical system using numerical simulation. Similarly, the critical damage parameter of the same material has been predicted by an entropy flow equation [18]. The irreversible entropy can be utilized to evaluate the reliability of a material or system accurately.

The first and second laws of thermodynamics can be applied to predict mechanical reliability, and statistical entropy plays an important role in estimating the fatigue life of components. The reliability of a Gatling gun’s automatic mechanism can be analyzed by combining the entropy generation value with neural networks, and the fault recognition rate can be improved effectively [63]. Zhang et al. [1] proposed a method combining mechanical stress and strain to characterize mechanical properties by applying statistical entropy. This method can be applied to reliability prediction for mechanical parts, such as connecting rods, rear axle housings, and vehicle half-shafts. Given the noise and frequency changes during mechanical performance changes (such as cracks in gears) in a statistical entropy test, dual-frequency and single-frequency entropies can detect the signals of different frequencies. Consequently, Radkowski et al. [66] applied the statistical entropy method to failure diagnosis. Furthermore, statistical entropy generation methods and theories have been applied to the hardware of mechanical parts and to software failure assessment. For example, a new entropy aging indicator has been proposed, and software aging has been estimated on the basis of multidimensional multiscale entropy [60,61]. Recently, information entropy is used in the fault diagnosis of rotating machinery [67,68,69], such as rolling bearings [67].

The significant investigations about the entropy generation analysis of mechanical failure are summarized in Table 2.

### 3.2. Methodologies for Defect Diagnosis

From its initial proposal to its subsequent evolution, the second law of thermodynamics has been proved to be effective. It has roughly evolved into two specified directions, including internal and surface entropy losses. Internal entropy is further deduced by considering the unit cycle energy and reliability-entropy.

First, the internal FFE generation of a metal sample can be obtained as [16,64]

(26) $$S=\int_{0}^{t_{f}}\left(\frac{W_{p}}{T}+\frac{k\Delta T^{2}}{T^{2}}\right)dt$$

Equation (26) shows the entropy generation that consists of mechanical dissipation due to plastic deformation and thermal dissipation due to heat conduction. The entropy generation owing to plastic deformation is far greater than the entropy generation owing to heat conduction under a low cycle process. Therefore, the latter is negligible, and Equation (26) can be simplified as

(27) $$S=\int_{0}^{t_{f}}\left(\frac{W_{p}}{T}\right)dt$$

where

(28) $$W_{p}=\rho CR_{\theta }$$

and *t* is time. This entropy formula takes into account temperature gradients, specific heat, and density. Al-6061-T6 and SS304 have been tested on the basis of Formula (27). The result indicated that entropy is constant in value and is not affected by other loadings and frequency [16]. Equation (27) has been further applied to the analysis of microstructure cracks appearing on electronic packaging substrates, landing pads, and TSV bodies [14]. The results showed that the entropy generation of the landing pad is 50% greater than that of the TSV body and thus indicated that the landing pad is easier to damage than the TSV body.

Traditional unit cycle energy $Q_{cyc}$ can be used as a property value for materials because the fatigue result for each material is a constant value [59]; that is,

(29) $$Q_{cyc}=-\frac{\rho C}{f}\frac{dT}{dt}$$

The unit cycle energy $Q_{cyc}$ can be linked to the FFE in the above formulas [70]:

(30) $$FFE=\frac{Q_{cyc}\cdot N_{f}}{T_{s}}$$

FFE determination can be employed to study the irreversible fatigue process of metal materials, such as AI7075-T6 and 1018 carbon steel [64]. Reliability-entropy has been developed from the popular sliding wear equation for frictional wear. The sliding wear formula, which considers wear coefficient, hardness pressure, normal load, and distance, is expressed as [7]

(31) $$w=\frac{kLx}{Har}$$

The correspondence between frictional wear and entropy production is expressed as

(32) dw∝dS

Considering the wear-time curve and entropy generation-time curve, we have

(33) $$\frac{dw}{dt}=\sum B\frac{ds}{dt}$$

Based on Equation (32), the reliability-entropy hypothesis, which has been proposed to establish the relationship between reliability and entropy, can be calculated as follows [7]

(34) Re(t)=1−∫0Td(t)Ddt=1−∫0Tdsdtdt

where

(35) $$\frac{ds}{dt}=\frac{W}{T}=\frac{F\times v}{T}$$

The reliability-entropy hypothesis described above can detect the reliability of metals, such as Al and brass [60].

Second, the energy loss of the material surface during fatigue aging is important. To monitor the critical defect value of mechanical structures in the aging process, a study has defined the entropy flow to the environment by calculating the temperature of the mechanical sample surface [18]:

(36) $${\dot{S}}_{e}=\frac{dS_{e}}{dt}$$

The heat flow to the environment can be expressed as

(37) $$q=h\left(T-T_{a}\right)$$

where T is the surface temperature, and $T_{a}$ is the ambient temperature. Then, the entropy flow rate to the air can be obtained as [18]

(38) $${\dot{S}}_{e}=\frac{h\left(T-T_{a}\right)}{T}=h\left(1-\frac{T_{a}}{T}\right)$$

The total entropy flow can be obtained in the integral of the entropy flow rate over time using the equation [18]

(39) $$S_{e}=\int_{0}^{t_{f}}\frac{h\left(T-T_{a}\right)}{T}dt$$

The relationship between standardized cyclic failure entropy and normalized cycle number can be expressed as [17]

(40) $$\frac{S_{e}}{S_{f}}≅\frac{N}{N_{f}}$$

where $S_{f}$ is the maximum entropy flow rate value, $N_{f}$ is the cyclic value of the final failure, and *N* is the cyclic number. The parameters of the critical defect of mechanical materials can be characterized and predicted using Equation (39). Meanwhile, the damage parameter (*D_N_*) has been provided as [70]

(41) $$D_{N}=-\frac{D_{\left(N_{f}-1\right)}}{\ln(N_{f})}\ln(1-\frac{N}{N_{f}})$$

The damage variable (*D*) can be given as

(42) $$D=f\left(\frac{S_{e}}{S_{f}}\right)$$

and further derived as

(43) $$D=\frac{1}{\ln(S_{f})}\ln(1-\frac{S_{e}}{S_{f}})$$

Therefore, the damage-entropy-time parameter is only related to time, and the entropy flow formula and critical defect value have been proposed accordingly [18]. Investigations to understand the defect characteristics of mechanical components with internal and/or surface entropy losses should be carried out in the future. Other low-cycle fatigue failure prediction models are available in the literature [63,64,65].

## 4. Conclusions

In this work, an overview is given of the development of methods for the evaluation of irreversible degradation based on the entropy generation. Two categories of methods are identified: damage analysis for electronic devices and defect diagnosis for mechanical components. The detailed derivation of the developed entropy generation formulas is discussed. It is highlighted that damage analysis entropy for electronic devices focuses on the heat generated by electronic devices. Meanwhile, the entropy generation theory in defect diagnosis for mechanical components focuses on mechanical deformation work. On the basis of the entropy generation studies reviewed, the following conclusions are made:

(1) The reliability prediction and defect diagnosis for electronic systems are focused on the influence of temperature gradient. In addition, the investigation on the relationship between power and entropy generation and the assessment of electronic system reliability is still in its early stages.
(2) The prediction of fatigue in mechanical components focuses on the influence of structural deformation by ignoring the influence of temperature gradient. Mechanical materials are mostly metal materials with good thermal conductivity during the entire fatigue failure; thus, their temperature change is minimal. Further investigations into the defect characteristics of mechanical components with internal and/or surface entropy losses should be carried out in the future.
(3) Unlike that of electronic and mechanical materials, the entropy generation of heat transfer systems focuses on temperature gradients and many fluid parameters, such as friction coefficient, Nusselt number, and Boit number. Moreover, the cooling system parameters and structural parameters of a heat exchange system are optimized on the basis of the second law of thermodynamics to improve system reliability.
(4) Care should be taken on the boundary conditions; radiative heat transfer is neglected in the vast majority of studies on entropy generation. This oversight is an important drawback because radiation dramatically affects most high power devices and plays an important role in high-density circuit design. Thus, the consideration of thermodynamic irreversibility due to radiative phenomena is necessary for future works, especially those on high density electronic and mechanical components.
(5) Further irreversible aging monitoring of electronic devices is expected to become a growing trend in the future for entropy generation methodologies. Power devices, sensors, and third-generation semiconductors should be considered in irreversible burn-in evaluation to improve their reliability.
(6) The research on the fatigue testing of metal materials should be expanded to ensure variety. Metal materials, such as stainless steel and Al, have been studied in a number of investigations, but few studies have focused on the fatigue analysis of other types of metal materials (e.g., Cu) based on entropy generation. In addition, the research on fatigue testing should progress to nonmetal materials with the second law of thermodynamics. Nonmetal materials for epoxy, such as Si, are widely used in electronic components, but related irreversible thermodynamic research theories and applications are limited.

## Figures and Tables

**Table 1:** Summary of investigations on electronic components.

Authors	Application Object	Highlights
Zimparov et al. [53]	Spiral bellows	- An equation of the performance evaluation criteria was developed to evaluate heat transfer enhancement techniques for entropy analysis.
Lai et al. [9]	SnPb solder joint	- The influence of entropy generation on the thermal transfer of a solder joint microstructure was presented.
Yang et al. [22]	Printed circuit board stack package	- The thermal optimization of stacked printed circuit boards was realized. - Entropy production is affected by the flow and temperature fields.
Zhang et al. [34] Cui et al. [10]	Die attach	- The cohesive zone method can predict interfacial stratification. - Entropy generation increases with increasing crack length.
Wang et al. [14]	Through-silicon via (TSV) mobile applications	- Fatigue analysis and prediction of TSV without any fitting data were studied. - Entropy generation was applied to the fatigue lifetime of mobile chips and full-chip TSV.
Shah et al. [51] Shah et al. [29]	Intel chip Electronic package	- Power distribution based on exergy analysis for future processor chips was analyzed. - An uneven coefficient was proposed to evaluate thermal performance.
Tian et al. [54]	A review of thermodynamic evolution	- The second law of thermodynamics was applied to a 1D structure, and recommendations for future applications were reviewed.
Aziz et al. [27]	Entropy generation in an asymmetrically cooled slab	- The total entropy generation rate depends on five dimensionless parameters. - Appropriate cooling parameters can result in the minimization of the total entropy.
Aziz et al. [28]	Classical and minimum entropy generation analyses	- Three different heat transfer coefficients with entropy were analyzed. - Regular and functionally graded materials were considered.
Torabi et al. [26]	Asymmetric cooling composite geometries	- Temperature distribution and local and total entropy generation were analyzed. - Three composite media, including composite walls, cylinders, and spheres, were studied.
Torabi et al. [50]	A thermal system with a solid structure	- The main solutions include accurate numerical methods, rough numerical methods, and software simulations.
Mohammadi et al. [55]	Cross laminate	- Thermodynamic entropy can predict the fatigue life of cross-layered plates.
Cuadras et al. [11]	Carbon film resistors	- Entropy is a more important indicator than resistance degradation in resistor research.
Cuadras et al. [12] Hsiao et al. [30]	Capacitor	- Entropy generation was affected by capacitance, geometry, and voltage. - A sensor for detecting entropy generation in a capacitor was presented.
Cuadras et al. [13]	Light emitting diodes (LEDs)	- The degradation entropy generation rate is independent of light parameters. - A threshold of entropy generation of end-of-life LEDs was proposed.
Lai et al. [9]	Pb37Sn63 solder joint	- A new damage indicator combines traditional damage parameters with entropy generation.

**Table 2:** Summary of the investigations on mechanical components.

Authors	Application Object	Highlights
Italyantsev et al. [60]	Mechanical parts	- The reliability of working mechanical parts can be predicted.
Tchankov et al. [61]	35 steel	- The fatigue life was predicted by calculating the hysteresis energy. - The method avoids the cyclic counting procedure and is not limited to high or low cycle fatigue.
Naderi et al. [16]	6061-T6 aluminum and 304 stainless steel (SS)	- The fatigue life of components was determined. - Irreversible entropy was verified as a degradation attribute.
Naderi et al. [17]	Al-6061 and SS 304	- The fatigue damage evolution with cyclic energy dissipation was determined. - The fatigue failure of components under torsion, bending, and tension-compression can be tested.
Amiri et al. [18]	Aluminum	- Low cycle bending fatigue on irreversible heat dissipation was studied. - Heat transferred from the surface area of the specimen to the surroundings was emphasized.
Eger et al. [62]	Electric machines	- Complex flow processes were analyzed with entropy in real alternator systems. - Heat exchange processes, including concerning fluid and heat transport, were optimized.
Ontiveros et al. [19]	Al 6061-T6 and SS 304 L	- The entropy accumulation of failure life is a constant value.
Slattery et al. [65]	Composite wall	- Evaluation of fresh crack surfaces based on macroscopic entropy was considered. - Macroscopic energy balance and macroscopic entropy inequality were considered.
Pan et al. [63]	Automatic mechanism of guns	- Entropy value was used as input to realize fault diagnosis. - The fault recognition rate can be improved with entropy.
Zhang et al. [1]	Connecting rod, vehicle axle	- The reliability prediction of structures can be realized.
Jang et al. [68]	Metal fatigue	- FFE was treated as a property of metal materials. - FFE was not affected by loading condition, frequency, and geometry.
Gidwani et al. [7]	Wear mechanics and system reliability	- The reliability-entropy hypothesis was applied to predict mechanical system reliability. - The degradation entropy generation theorem was considered.
Radkowski et al. [66]	Gear crack	- The entropy method was used for failure diagnosis. - Single-frequency entropy can read the signals of frequency differences.

## Nomenclature

S	Entropy generation, J kg^−1^ K^−1^
Q	Heat, J
T	Temperature, K
$E_{\mathrm{in}}$	Input energy, J
$\dot{w}$	Heat dissipated at a certain temperature, J/s
W	Light output energy, J
$E_{irr}$	Irreversible energy, J
P	Power
t	Time, s
U	Voltage drop, V
I	Electric current, A
λ	Wavelength, nm
R	Resistance
$S_{e}$	External entropy generation
$S_{i}$	Internal entropy generation
$Q_{e}$	Heat dissipated into the environment, J
$K_{\lim}$	Limit constant
$C_{p}$	Capacitance of pyroelectric material
η	Absorption coefficient of radiation
A	Electrode area, m^2^
$T_{f}$	Final temperature, K
$T_{i}$	Initial temperatures, K
ψ	Exergy
G	Specific enthalpy, J/kg
R	Thermal resistance
a	Ambient
c	Case
CS	Case-to-sink
FoM	Related to the figure of merit
in	Input
j	Junction
$\eta_{∐}$	Second law coefficient
$Z_{power}$	Uniformity coefficient
T(X)	Temperature-dependent distance, K
$N_{f}$	Total number of cycles to failure
F	Driving force, N
*P*	Pressure
T(t)	Temperature-dependent time, K
$J_{p}$	Heat flux through the boundary
W(t)	Work by deformation
V	Solder volume
kT	Thermal energy
D	Diffusivity
N	Avogadro’s number
M	Mass rate
$\vec{q}$	Heat flux
gradT	Temperature gradient
$t_{f}$	Failure time
C	Heat capacity
h	Convective heat transfer coefficient
$D_{N}$	Damage parameter
$Q_{cyc}$	Unit cycle energy, J
k	Wear coefficient
$H_{ar}$	Hardness pressure
L	Normal load
B	(dw/ds) for a particular process
jc	Junction-to-case
ja	Junction-to-ambient
p	Related to processor
out	Outlet
S	Sink
sa	Sink-to-ambient
